# MMP11 as a Prognostic Indicator for Inverted Papilloma Histologic Grade and Recurrence

**DOI:** 10.1002/lary.70072

**Published:** 2025-08-22

**Authors:** Kush Panara, Jadyn Wilensky, Alan D. Workman, David K. Lerner, Alexandria L. Irace, Jennifer E. Douglas, James N. Palmer, Nithin D. Adappa, Jalal Jalaly, Charles C. L. Tong, Noam A. Cohen, Michael A. Kohanski

**Affiliations:** ^1^ Department of Otorhinolaryngology‐Head and Neck Surgery Perelman School of Medicine, University of Pennsylvania Philadelphia Pennsylvania USA; ^2^ Department of Otolaryngology Massachusetts Eye and Ear/Harvard Medical School Boston Massachusetts USA; ^3^ Department of Otolaryngology University of Miami Miami Florida USA; ^4^ Monell Chemical Senses Institute Philadelphia Pennsylvania USA; ^5^ Department of Pathology Perelman School of Medicine, University of Pennsylvania Philadelphia Pennsylvania USA; ^6^ Department of Otolaryngology‐ Head and Neck Surgery Lenox Hill Hospital/Long Island Jewish Hospital New York New York USA; ^7^ Corporal Michael J. Crescenz VA Medical Center Philadelphia Pennsylvania USA

**Keywords:** inverted papilloma, matrix metalloproteinase‐11, recurrence

## Abstract

**Objectives:**

Inverted papilloma (IP) is a benign, but locally aggressive sinonasal tumor with a high recurrence rate and potential for malignant transformation into squamous cell carcinoma ex‐IP (SCC). Currently, no reliable biomarkers exist to predict recurrence or severity. Matrix metalloproteinase‐11 (MMP11) has been implicated in tumor progression and extracellular matrix remodeling in various malignancies and has been shown to be progressively upregulated in IP transformation. This study investigates the relationship between MMP11 expression and IP histologic severity and recurrence risk.

**Methods:**

Patient demographics and tumor characteristics were collected retrospectively at a tertiary care center from 2008 to 2023. Patient specimens were categorized into normal sinus tissue (NST), IP without dysplasia (IPND), IP with severe dysplasia (IPSD), and SCC. Immunohistochemical staining for MMP11 was performed, and quantitative analysis was conducted using DAB‐Quant software.

**Results:**

A total of 52 patients were included, with a mean follow‐up of 68.8 months. MMP11 expression was significantly elevated in IPSD and SCC compared to IPND (36.7% and 36.5% vs. 21.1%, *p* = 0.02 and *p* = 0.01). Patients with recurrence exhibited higher MMP11 expression in their initial resection specimens compared to non‐recurrent cases (31.1% vs. 18.1%, *p* < 0.01). High MMP11 expression (> 50%) was associated with a shorter time to recurrence (17.5 vs. 32 months, *p* = 0.03), independent of histologic grade.

**Conclusion:**

MMP11 expression correlates with increased IP histologic grade, risk of recurrence, and shorter time to recurrence. Elevated MMP11 expression may identify patients at higher risk for recurrence, allowing for targeted surveillance strategies and treatment options.

**Level of Evidence:**

3.

## Graphical abstract

Matrix metalloproteinase‐11 (MMP11) expression increases with inverted papilloma (IP) histologic grade and is significantly higher in patients who develop recurrence. Quantitative immunohistochemistry revealed that elevated MMP11 predicts both increased recurrence risk and shorter time to recurrence, independent of dysplasia. These findings highlight MMP11 as a promising prognostic biomarker to guide personalized surveillance strategies in IP.

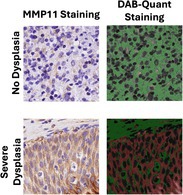

## 1 Introduction

Inverted papilloma (IP) is a benign but locally aggressive sinonasal tumor characterized by the proliferation of respiratory epithelium with downward growth into the underlying stroma while maintaining an intact basement membrane [1]. Despite its benign nature, IP presents significant clinical challenges due to its high recurrence rate, ranging from 15% to 25% [2], and its potential for malignant transformation into squamous cell carcinoma ex‐IP (SCC), which occurs in approximately 5%–15% of cases [3]. Current treatment relies on surgical resection with attention to the attachment site, followed by long‐term surveillance [4]. While a variety of molecular mechanisms have been investigated, there are currently no established biomarkers to reliably predict recurrence or transformation, making patient management challenging and necessitating frequent endoscopic surveillance.

One area of increasing interest in tumor biology is the role of matrix metalloproteinases (MMPs), a family of zinc‐dependent endopeptidases involved in extracellular matrix (ECM) remodeling and tumor progression [5]. Among them, matrix metalloproteinase‐11 (MMP11), also known as stromelysin‐3, is unique in its activation mechanism and substrate specificity [6]. Unlike most MMPs that degrade ECM proteins in the extracellular space, MMP11 is activated intracellularly and modifies the tumor microenvironment, promoting cancer cell survival, invasion, and immune evasion [6]. Elevated MMP11 expression has been associated with increased tumor aggressiveness and poor prognosis in several malignancies, including breast, gastric, lung, pancreatic, and oral cancers [6, 7, 8, 9].

In the context of IP, emerging evidence suggests that MMP11 plays a role in disease progression. Prior work has demonstrated that MMP11 expression is increased through the neoplastic progression stages of IP degeneration to invasive SCC, with the highest levels observed in cases exhibiting severe dysplasia and SCC transformation [10]. Additionally, recent work has identified MMP11 as a mediator of epithelial migration and invasion in IP [11]. However, its potential role in predicting recurrence remains unclear. This study aims to assess MMP11 expression across different histologic subtypes of IP and evaluate its ability to predict recurrence.

## 2 Materials and Methods

### 2.1 Patient Specimens

Patients who underwent surgery and excision for IP from 2008 to 2023 at our institution were identified, and informed consent was obtained. Approval from the University of Pennsylvania Institutional Review Board was obtained prior to the study. Specimens of varying histology were selected and categorized into the following: (1) Normal sinus tissue (NST), (2) IP without dysplasia (IPND), (3) IP with severe dysplasia (IPSD), (4) SCC ex‐IP. Patients diagnosed with recurrence after primary surgery at our institution were identified, and specimens from the index surgery (prior to recurrence) were collected. Patients were excluded if they had incomplete medical records, initial surgery at an outside institution, or did not have tumor tissue available.

### 2.2 Immunohistochemistry

An independent pathologist evaluated tumor slides and confirmed the degree of dysplasia or invasion. Immunohistochemistry (IHC) for MMP11 (Clone SN74‐08, Invitrogen MA5‐32285, 1:200 dilution) staining was performed on five‐micron sections of formalin‐fixed paraffin‐embedded tissue samples on a Leica Bond‐IIITM instrument using the Bond Polymer Refine Detection System (Leica Biosystems DS9800). Heat‐induced epitope retrieval was carried out using ER2 solution (Leica Microsystems AR9640) for 20 min. After incubation with the primary antibody, the slides were treated with a visualization reagent containing a secondary antibody for 30 min at room temperature, followed by development using 3,3′‐diaminobenzidine (DAB) chromogen for 1 min. Counterstaining was performed with Mayer's hematoxylin. Slides were thoroughly washed between each step with bond wash buffer or distilled water. Following dehydration and mounting, the slides were digitally scanned for subsequent image analysis.

Classification of IHC expression was obtained by quantitative analysis of MMP11 staining. DAB‐Quant software was employed to objectively quantify DAB‐stained areas based on pixel intensity and staining distribution [12]. Twenty‐five representative epithelial regions per slide were randomly selected, and the mean staining intensity was calculated. A background threshold was established using control specimens that had undergone the same staining protocol in the absence of primary antibody exposure. The percentage of positively stained areas was computed and analyzed to assess differences between histologic groups as a reproducible and unbiased quantification of MMP11 expression in tissue samples.

### 2.3 Statistical Analysis

Statistical analysis was performed using GraphPad Prism version 10.0.0 for MacOSx (Boston, Massachusetts USA). Statistical significance was determined using Student's *t*‐test or ANOVA followed by post hoc analysis. Time to recurrence was calculated by the Kaplan–Meier method. A *p*‐value < 0.05 was considered statistically significant.

## 3 Results

### 3.1 Patient Characteristics

A total of 52 patients who underwent resection for IP were included in the study. The cohort consisted of 38 males and 14 females, with a mean age of 60.9 years (range: 25–86 years). The mean follow‐up duration was 68.8 months. Of these patients, 17 specimens represented the primary tissue specimens of patients with subsequent recurrent disease. No differences in MMP11 expression between age, site of attachment (SOA), or gender were seen. Table 1 summarizes the patient demographics, tumor site distribution, and histologic classifications.

**TABLE 1:** Patient characteristics.

Histology		*p* < 0.03
NST	7 (13.4%)	
IPND	29 (55.8%)	
IDSD	7 (13.5%)	
IPSCC	9 (17.3%)	
Age	60.9 years	*r* = 0.06, *p* = 0.64
Sex		*p* = 0.34
Male	38 (73.1%)	
Female	14 (26.9%)	
SOA		*p* = 0.77
Maxillary	20 (38.5%)	
Ethmoid	5 (9.6%)	
Sphenoid	5 (9.6%)	
Frontal	11 (21.2%)	
Septum	6 (11.5%)	
Unknown	5 (9.6%)	
Follow up	68.8 months	

Abbreviations: IPND, inverted papilloma without dysplasia; IPSD, inverted papilloma with severe dysplasia; NST, normal sinus tissue; *r*, Pearson's coefficient; SCC, squamous cell carcinoma ex‐IP; SOA, primary site of attachment.

### 3.2 Association of MMP11 Expression With Histologic Grading

**FIGURE 1:**
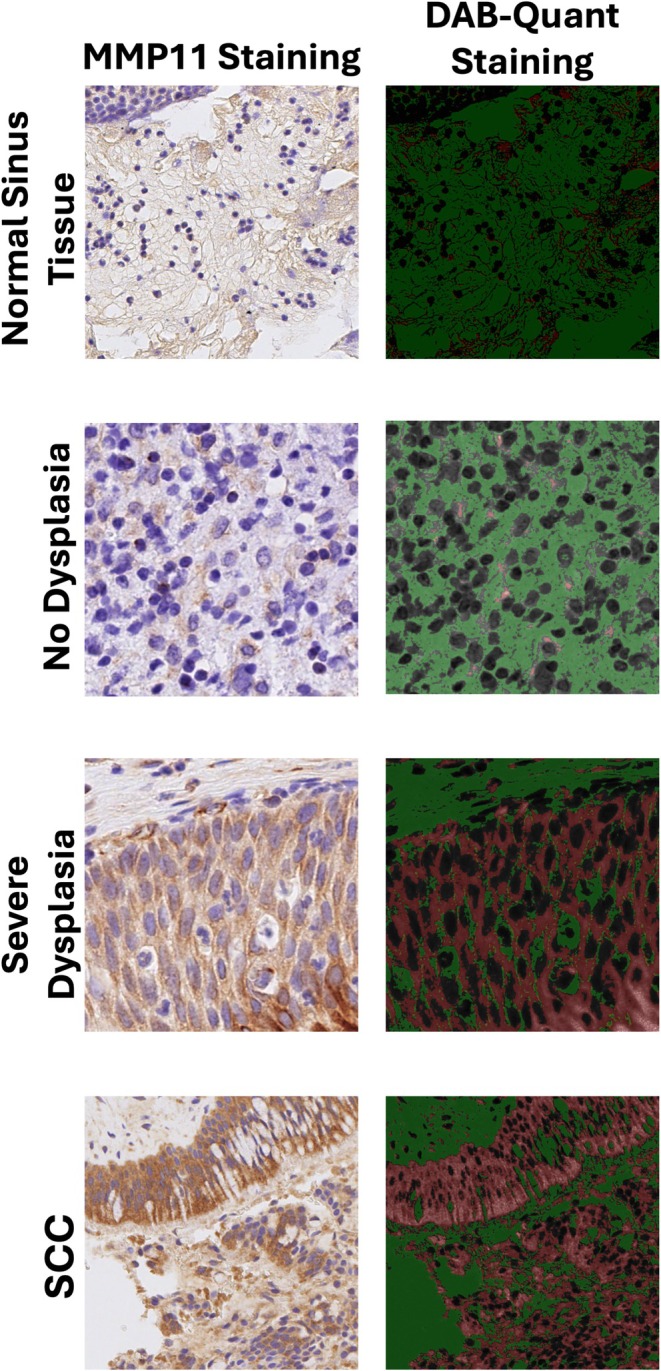
Immunohistochemistry of inverted papilloma (IP) specimens. Representative images of immunohistochemistry staining of IP specimens with MMP11 among different histologic grades (Left column). DAB‐Quant Staining shows representative images post‐processing through the DAB‐Quant program. Green demonstrates background pixels, black is non‐stained tissue, red is positive stained pixels for DAB, representing areas positive for MMP11 (Right column). MMP11, matrix metalloproteinase‐11, SCC, squamous cell carcinoma ex‐IP. [Color figure can be viewed in the online issue, which is available at www.laryngoscope.com]

**FIGURE 2:**
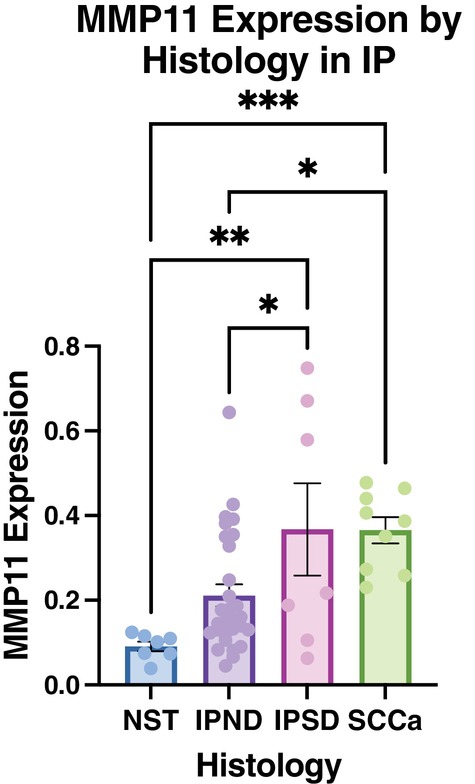
MMP11 correlates with histologic grade. IP and NST specimens underwent IHC with MMP11. IPSD and SCC had significantly higher MMP11 expression compared to NST (36.7% and 36.5% vs. 9.1%, *p* < 0.01 and *p* < 0.001). In IPSD and SCC, MMP11 expression was significantly higher compared to IPND (36.7% and 36.5% vs. 21.1%, *p* = 0.02), suggesting that MMP11 is related to higher tumorigenicity. * < 0.05, ** < 0.01, *** < 0.001. [Color figure can be viewed in the online issue, which is available at www.laryngoscope.com]

There is upregulation of gene expression of MMP11 as IP undergoes malignant degeneration [10]. To correlate these findings at the protein level, MMP11 expression was analyzed in IP tumor specimens across different histologic subtypes (Figure 1), including NST (*n* = 7), IPND (*n* = 29), IPSD (*n* = 7), and SCC (*n* = 9). IHC staining demonstrated that among IP, both IPSD and SCC showed significantly higher MMP11 expression as compared to IPND (36.7% and 36.5% vs. 21.1%, *p* = 0.02, Figure 2). MMP11 expression was also significantly elevated in IPSD and SCC compared to NST (36.7% and 36.5% vs. 9.1%, *p* < 0.01 and *p* < 0.001, Figure 2). Although there was a trend toward increased MMP11 expression in IPND compared to NST, the results did not reach significance (21.1% vs. 9.1%, *p* = 0.07). No differences in MMP11 expression were observed when specimens were stratified based on age, sex, or primary site of attachment.

### 3.3 MMP11 Expression and Recurrence Risk

**FIGURE 3:**
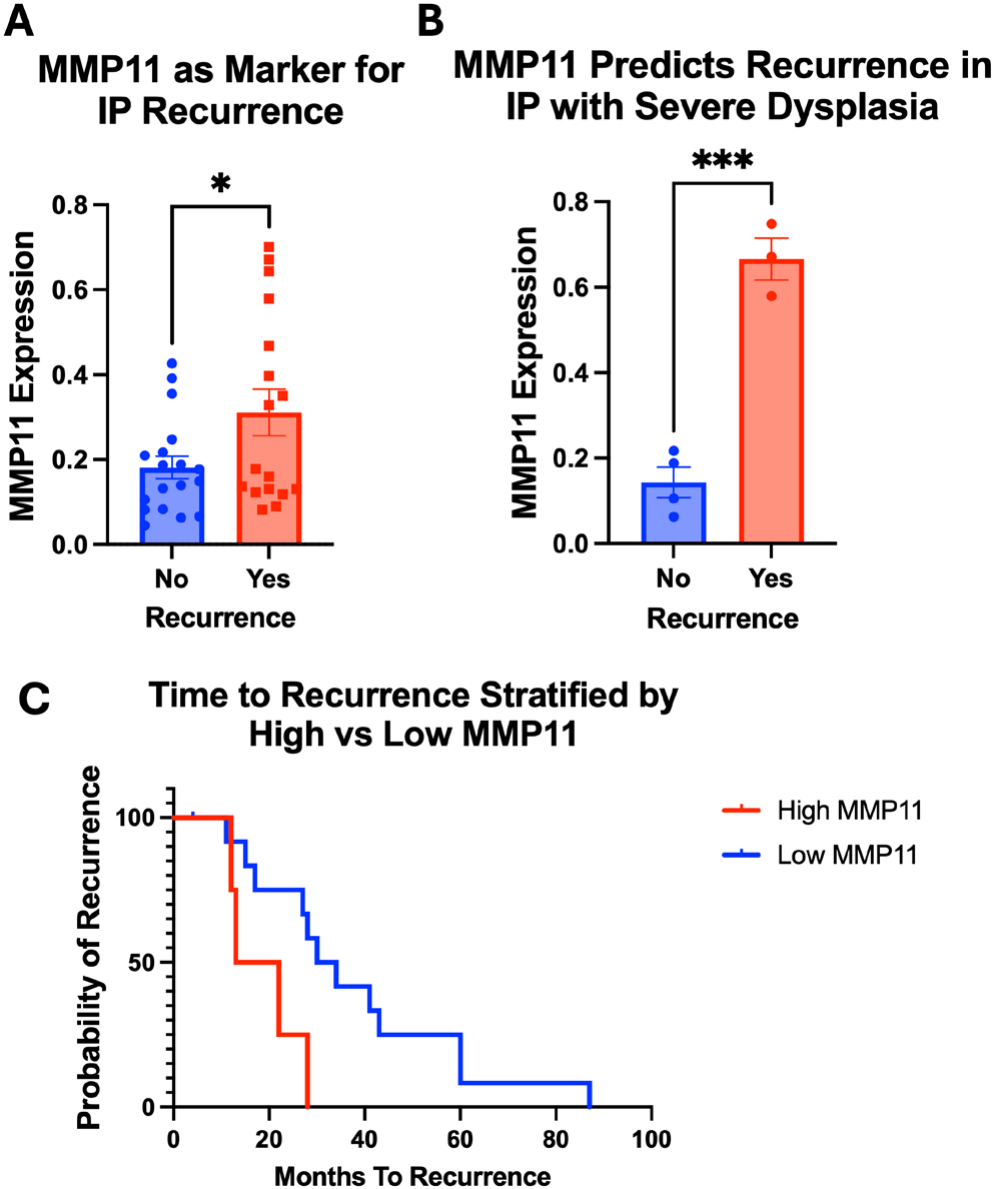
MMP11 predicts recurrence and is associated with shorter time to recurrence. (A) Patients with IP recurrence demonstrated significantly elevated MMP11 expression in their tumors from the primary surgery compared to those without IP recurrence (31.1% vs. 18.1%, *p* < 0.01, A). (B) Subgroup analysis in primary IPSD samples shows IP recurrence is exclusively associated with elevated MMP11 (66.6% vs. 14.3%, ***p < 0.001, B). (C) Among patients who had an IP recurrence, primary tumors with elevated MMP11 (> 50%) were associated with a significantly shorter time to recurrence as compared to primary tumors with low MMP11 (< 50%) (17.5 vs. 32 months, *p* < 0.01, C). [Color figure can be viewed in the online issue, which is available at www.laryngoscope.com]

Post‐resection surveillance for IP is important given significant recurrence rates; however, there are no markers to predict recurrence [13, 14]. We next evaluated the utility of histologic MMP11 expression to predict recurrence. MMP11 expression was compared in patients who experienced future recurrence (*n* = 17) versus a subset of those who did not have recurrence (*n* = 18). One patient with IPSD was excluded due to loss of follow‐up after initial surgery. Patients with IP recurrence demonstrated significantly elevated MMP11 expression in their tumors from the primary surgery compared to those without IP recurrence (31.1% vs. 18.1%, *p* < 0.01, Figure 3A). Of note, higher levels of MMP11 at the time of primary surgery were significantly higher in IPSD with recurrence compared to IPSD patients without recurrence (66.6% vs. 14.3%, *p* < 0.001, Figure 3B).

### 3.4 Time to Recurrence Analysis

Given the association of MMP11 with recurrence and histopathologic severity, we next assessed if MMP11 was associated with time to recurrence. Patients with elevated MMP11 expression (> 50%) had a significantly reduced mean time to recurrence compared to those with lower expression levels (< 50%) (17.5 vs. 32 months, *p* = 0.03, Figure 3C). Importantly, time to recurrence did not significantly differ between histologic subtypes, suggesting that MMP11 expression may function as an independent prognostic factor for time to recurrence.

## 4 Discussion

In this study, we demonstrate that MMP11 expression on IHC staining of primary IP tumor resections is significantly associated with histologic grade and recurrence risk. This study provides evidence for the role of MMP11 as a prognostic biomarker in IP, reinforcing its potential utility in predicting higher grade pathology as well as the risk of recurrence and time to recurrence.

The progressive upregulation of MMP11 expression as IP transforms into SCC has been previously noted in gene expression studies [10]. In general, the breakdown and remodeling of the ECM are essential steps for tumor cells to invade neighboring tissue and metastasize to distant sites [5]. MMPs, a family of zinc‐dependent proteolytic enzymes, are central to this process due to their ability to degrade ECM proteins, making them key players in tumor development. Recent work has described a role for MMP11 in epithelial migration specifically in IP epithelial cells, suggesting a role for MMP11 in tumor microenvironment remodeling, which is crucial for malignant transformation [11]. This is in line with the observed increased expression of MMP11 in cases with severe dysplasia and SCC, again highlighting that MMP11 may have a role in the breakdown of ECM proteins, facilitating epithelial migration and invasion. While MMP11 expression significantly increases as IP progresses in severity from no dysplasia, there was no significant difference in MMP11 expression between IPSD and SCC. This may be due to limited sample sizes or the involvement of other mechanisms independent of MMP11 that contribute to the final transformation from IPSD to SCC.

Although recurrence rates have improved with endoscopic surgical techniques and attachment‐oriented resection [15, 16], significant recurrence risk remains and patients with IP require close long‐term surveillance [1, 4]. Surveillance relies on endoscopy and/or imaging, but can be difficult in challenging anatomical areas and there are currently no known prognostic biomarkers for IP recurrence. One of the most notable findings of our study is the association between MMP11 expression and recurrence rate and timing. Patients with elevated MMP11 (> 50%) experienced both increased risk of recurrence as well as a significantly shorter time to recurrence compared to those with lower levels of MMP11. Importantly, our results suggest that MMP11 expression may serve as a predictive factor for recurrence independent of dysplasia. Given the lack of established biomarkers for recurrence in IP, our findings highlight's MMP11's potential role in risk stratification and personalized patient surveillance.

In addition to MMP11, several other biomarkers have been investigated in the context of IP recurrence and malignant transformation, though there is inconsistency in the literature and none have been validated for clinical use. Notably, overexpression of p53 has been associated with higher histologic grade and transformation to squamous cell carcinoma (SCC), suggesting its potential role in identifying more aggressive IP subtypes [17, 18]. Similarly, epidermal growth factor receptor (EGFR) mutations have been implicated in both the pathogenesis and malignant potential of IP, with studies indicating that EGFR exon 20 mutations may represent a distinct molecular subtype with increased risk for recurrence [19, 20]. Additionally, human papillomavirus (HPV) infection has been inconsistently reported in IP and SCC ex‐IP, with some suggesting an association with dysplasia, while others find the presence of HPV DNA only in a minority of IP samples [21, 22, 23]. These findings highlight the heterogeneity of IP at the molecular level and underscore the complexity of its progression. The lack of a unified pathophysiologic mechanism or reliable biomarker emphasizes the need for further study of pathways involved in epithelial invasion, immune evasion, and extracellular matrix remodeling's. MMP11's association with both histologic grade and recurrence supports its potential role in this landscape and may reflect a convergent downstream effector in IP progression. Future work may benefit from integrating MMP11 with these additional markers into a multiparametric risk model for better prognostication. Furthermore, investigating the interaction of MMP11 with other components of the tumor microenvironment may identify novel therapeutic targets for preventing malignant transformation and recurrence. Preclinical models should also be leveraged to explore MMP11‐targeted interventions, potentially opening new avenues for adjuvant therapy in high‐risk patients.

An important future step is evaluating the utility of circulating MMP11 in patient serum or potentially from mucus or nasal brushings as a non‐invasive biomarker for recurrence. Circulating MMP11 has been explored in other malignancies with promising results [24, 25]. While our study assessed tissue‐based MMP11 expression, serum‐based assays could provide a more accessible and dynamic measure of disease status. Future studies should assess whether serum MMP11 levels correlate with outcomes and recurrence in IP. In addition, MMP11 is likely one of many factors contributing to the complex process of IP malignant degeneration. This study lays the groundwork for further investigation of other progressively upregulated genes as IP degenerates to SCC. A panel of these markers used in combination may be able to predict transformation and recurrence with high accuracy.

### 4.1 Limitations

Despite the promising nature of our findings, certain limitations must be acknowledged. The retrospective design of our study introduces potential selection bias, and the sample size, particularly in subgroup analyses, is relatively small. Furthermore, the evaluation of histologic grade remains subjective and is dependent on the evaluated specimen. Underlying tumor heterogeneity may lead to areas of dysplasia or transformation not being sampled adequately. We opted to use objective IHC quantification to limit subjectivity using the open‐source DAB‐Quant program. Although we applied the same parameters to all specimens and used 25 selections to minimize inter‐slide variability, there is a chance that the calculated MMP11 expression differs from the true expression. Additionally, while IHC quantification of MMP11 provides valuable insights, complementary techniques such as RNA sequencing or proteomic profiling could further delineate its role in IP pathogenesis. Larger, prospective, multicenter studies are necessary to validate our findings and assess the generalizability of MMP11 as a biomarker across diverse patient populations.

## 5 Conclusion

Patients who undergo resection of IP currently require long‐term surveillance. No prognostic markers currently exist for IP to help triage patients' risk for recurrence and transformation. MMP11 is a marker that has been associated with IP migration and invasion. Here we show that elevated MMP11 correlates with histologic grade and is associated with a shorter time to recurrence independent of histology, highlighting the role of MMP11 as a potential prognostic biomarker in IP.

## Conflicts of Interest

The authors declare no conflicts of interest.

## Data Availability

The data that support the findings of this study are available from the corresponding author upon reasonable request.
